# AtRTPrimer: database for Arabidopsis genome-wide homogeneous and specific RT-PCR primer-pairs

**DOI:** 10.1186/1471-2105-7-179

**Published:** 2006-03-30

**Authors:** Sangjo Han, Dongsup Kim

**Affiliations:** 1 Department of BioSystems, Korea Advanced Institute of Science and Technology, 373-1 Guseong-dong, Yuseong-gu, Daejeon 305-701, Republic of Korea

## Abstract

**Background:**

Primer design is a critical step in all types of RT-PCR methods to ensure specificity and efficiency of a target amplicon. However, most traditional primer design programs suggest primers on a single template of limited genetic complexity. To provide researchers with a sufficient number of pre-designed specific RT-PCR primer pairs for whole genes in Arabidopsis, we aimed to construct a genome-wide primer-pair database.

**Description:**

We considered the homogeneous physical and chemical properties of each primer (homogeneity) of a gene, non-specific binding against all other known genes (specificity), and other possible amplicons from its corresponding genomic DNA or similar cDNAs (additional information). Then, we evaluated the reliability of our database with selected primer pairs from 15 genes using conventional and real time RT-PCR.

**Conclusion:**

Approximately 97% of 28,952 genes investigated were finally registered in *AtRTPrimer*. Unlike other freely available primer databases for *Arabidopsis thaliana*, *AtRTPrimer *provides a large number of reliable primer pairs for each gene so that researchers can perform various types of RT-PCR experiments for their specific needs. Furthermore, by experimentally evaluating our database, we made sure that our database provides good starting primer pairs for Arabidopsis researchers to perform various types of RT-PCR experiments.

## Background

In the post-genome era, microarray technology becomes a powerful tool for global gene expression profiling. However, some genes show significant variability in their expressions. These observed differences should be validated through more accurate tools. In addition, microarray methodology can not credibly monitor the low levels of expression from certain genes such as, transcription factors [1]. Quantitative RT-PCR methods (*i.e*. real-time RT-PCR) have been adopted to overcome the stated drawbacks [2]. For researchers, RT-PCR is useful and, moreover essential, for accurately measuring quantitative transcriptional levels of particular genes.

Primer design is a critical step in all kinds of RT-PCR methods to guarantee specificity and efficiency of a target amplicon. However, most traditional primer design programs suggest primers on a single template of limited genetic complexity [3]. Although several online RT-PCR primer databases have been established as repositories for empirically validated primer sequences submitted by researchers [4], unfortunately these databases contain primers for only a few hundred genes at present. Therefore, it is often necessary to design the primer pairs for genes of interest by trial and error. To overcome the disadvantage of online repositories of empirically validated primers, Wang and Seed [5] made genome wide primer pairs database for real-time RT-PCR called PrimerBank. This database was designed for the inexpensive and common types of real-time PCR relying on fluorescent detection of amplified DNA by sequence non-selectively dyes, such as SYBR Green I. Through hashing technique and high filter stringency, they obtained specific primers on all known coding genes in human and mouse. However, although this database is readily usable for real-time RT-PCR, it can not be applied to other useful types of RT-PCR, *i.e*., conventional multiplex PCR. In addition, there is no consideration of genomic DNA contamination.

In this work we aim to construct a genome wide RT-PCR primer database of specific primer pairs for all annotated Arabidopsis genes which can be used for the various types of RT-PCR experiments, such as conventional RT-PCR and real-time RT-PCR. To do this, we enriched the primer-pair resources using various information and selection criteria such as similar gene list of each gene and the nearest neighbor joining thermodynamics threshold [6]. In addition, we added information on all the potential amplicons that can be produced by a target gene's primer-pair from its corresponding genomic DNA or its similar genes (*i.e*. alternative splicing forms). These types of information will help a researcher to interpret unexpected amplicons of PCR result more easily.

## Construction and content

### Arabidopsis sequences

We used the sequences of flowering plant *Arabidopsis thaliana*, an important model system in plant biology. The Arabidopsis Genome Initiative reported the complete sequences of *Arabidopsis thaliana *genome [7]. All mRNAs including known alternative splicing forms and their corresponding genomic DNAs were downloaded from TIGR [8] and were used for generating candidate primers for a set of target genes, obtaining the exon positions, and performing BLAST searches. For clarity, we emphasize that we use the following terms, 'gene', 'cDNA' and 'mRNA', as the same meaning throughout the text. They all indicate the unique cDNA sequences.

### Procedures

**Figure 1:**
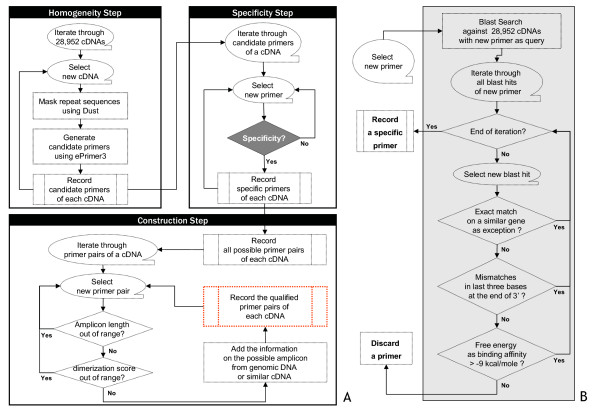
**Overall scheme for construction of *AtRTPrimer *(A) and flowchart for the decision for primer specificity (B)**. The *AtRTPrimer *was created through the following three steps: homogeneity, specificity, and construction steps. In homogeneity step, we generated the candidate primers from each of simple repeats-masked cDNA sequences using Dust program and ePrimer3, and then temporarily recoded. They have similar properties such as length, melting temperature and GC content. In the specificity step, we checked whether each primer of each cDNA is specific against 28,592 cDNAs. Then, we temporarily recoded specific primers of each cDNA. Right panel (B) shows how to make decision on the specificity of a primer of a cDNA. For that decision, first we ignored exact match for a primer of a cDNA to its similar cDNAs. This exception was reconsidered in construction step. Second, we checked whether or not there are mismatches in last three bases at the end of 3'. Third, we checked whether the binding affinity between a primer and a blast hit is greater than the free energy threshold. We decided that a primer had no specificity if one of all blast hits of the primer does not satisfy these three conditions sequentially. In the construction step, we filtered out the unqualified primer pairs from all possible primer pairs generated by specific primers of each cDNA. After filtering, we added the information on the possible amplicons from a genomic DNA or a similar cDNA to the remaining primer pairs (see Additional file 1 and Additional file 2)

We considered homogeneous physical and chemical properties of each primer (homogeneity) of a gene, non-specific binding against all other known genes (specificity), and other possible amplicons from its corresponding genomic DNA or similar cDNA (additional information) to construct *AtRTPrimer*. The entire procedure is shown schematically in Figure 1.

#### Homogeneity step

For the homogeneous primer selection (Figure 1-A), we first masked the simple repeat sequences of each cDNA using Dust program [9] to avoid selecting a primer from low-complex region. Then, we generated all possible primers from the masked cDNA sequences using the ePrimer3 program which is one of the EMBOSS packages [10]. The ePrimer3 parameters used for homogenous primer selections are the followings: melting temperature (Tm) is 66–70°C, primer length 22–25 bp, GC clamp (G or C at 3'end), primer-dimerization (PA ≤ 8, PE ≤ 3), and GC content 40–60%. Other ePrimer3 parameters were kept in their default values. After generating primers by ePrimer3 with the given parameters, we then excluded the primers of which GC % of half length is greater than a threshold percent. We applied that restriction so that we did not recruit primers of which most of the content of C or G is laid only in the region of 3' end or only in the region of 5' end.

#### Specificity step

We checked whether or not a primer of each cDNA is specific using BLAST search and three conditions. We performed BLAST search against 28,952 cDNAs using BLAST 2.0 program [11] with a primer as query and the parameters suggested by NCBI; when a small size of DNA such as a primer (*i.e*. typical size: 18–30) is aligned, the option of word size is 7 and e-value 1000 [12]. Because a query sequence size is small, there are typically many BLAST hits for most candidate primers. For the decision of primer specificity, we first ignored exact match for a primer of a cDNA to its similar cDNAs (called "condition 1"). Later, this exception was reconsidered in construction step (see Additional file 2). Second, we checked whether or not there are mismatches in the last three bases at the 3' end (called "condition 2"). Third, we checked whether or not the binding affinity between a primer and blast hit is greater than -9 kcal per mole as the free energy threshold (called "condition 3"). We decided that a primer has no specificity if there is a case where one of the blast hits does not meet these three exempting conditions sequentially (Figure 1-B). The purposes of having condition 1 and condition 2 are to rescue some primers among candidate primers that are otherwise discarded if we only consider condition 3 (free energy threshold).

For the condition 1, we made the list of genes similar to each target gene (see Additional file 2). The similar gene list (SGL) was used for obtaining more candidate primers from a target gene. Suppose that one target gene has more than two alternative splicing forms or is a member of gene family with very high sequence similarity. In that case, most candidate primers of a target gene will disappear unless we consider this condition. The condition 2 also gives a target gene a chance to have more candidate primers. The DNA polymerase can not react efficiently if last few residues at the 3' end of a primer do not match its template properly regardless of specificity in 5' end region. This characteristic has been utilized for PCR-based physical mapping technology, demonstrating that the polymerase does not work efficiently when the last two residues at the 3' end do not match its template, producing no amplicon [13]. In case of condition 2, the mismatches of last three base pairs were used as the threshold.

For the last condition, we calculated the Gibbs free energy as a measure for the non-specific binding affinity between a candidate primer and its corresponding BLAST hits. A statistical analysis of *in vivo *activities in more than 1000 experiments with antisense oligonucleotides was reported [14]. This work concluded that the values (ΔG°_37_) for self-interaction should be ≥-8 kcal/mol for inter-oligonucleotide pairing and ≥-1.1 kcal/mol for intra-molecular pairing for increasing the proportion of active antisense oligonucleotides by as much as 6-fold. We regarded inter-oligonucleotide pairing as the pairing between a candidate primer and its corresponding BLAST hit, and then calculated the free energy (ΔG°_62_) at 62°C between them by using the equation: ΔG°_62 _= ΔH° - TΔS°, where T is a temperature in kelvin and ΔH° and ΔS°, parameters for enthalpy and entropy, respectively. The parameters of "unified" oligonucleotide nearest-neighbor thermodynamics were used for calculation [6]. To obtain more candidate primers, we used the somewhat more generous cutoff, -9 kcal/mole as threshold, lower than free energy suggested in antisense statistical analysis.

#### Construction step

We filtered out the unqualified possible primer pairs as follows: Primer pairs with amplicon sizes between 100 and 800 bp were removed first. Second, if their dimerization scores are out of the following ranges: PA ≤ 8 and PE ≤ 3, the primer pairs were rejected. After filtering out the unqualified primer pairs, we added to the remaining primer pairs the information on possible amplicons from both genomic DNA and similar genes (see Additional file 1 and Additional file 2). The reason why we considered these types of information is as follows: We could obtain the best PCR results when we use a total RNA as template not including any contaminants such as a genomic DNA. However, the genomic DNA could not be completely removed from a total RNA, especially for plant. In addition, although it is ideal that one primer pair should be designed to produce only one amplicon, this requirement is often difficult for genes with many similar genes (*i.e*. in most cases, splicing variants). Our database included more candidate primers by allowing a primer pair to produce more than one amplicon. Finally, those qualified primer pairs with additional information on possible amplicons from a genomic DNA and similar cDNAs were imported to a MySQL database installed on a Linux server.

### Implementation

We implemented our procedures (Figure 1) in a program called *uniPrimer *using BioPerl 1.xx [15], EMBOSS 2.xx and MySQL 3.xx with 28,952 of total genes in *Arabidosis thaliana *to make the *AtRTPrimer *database.

## Utility and discussion

### Statistics of AtRTPrimer

**Figure 2:**
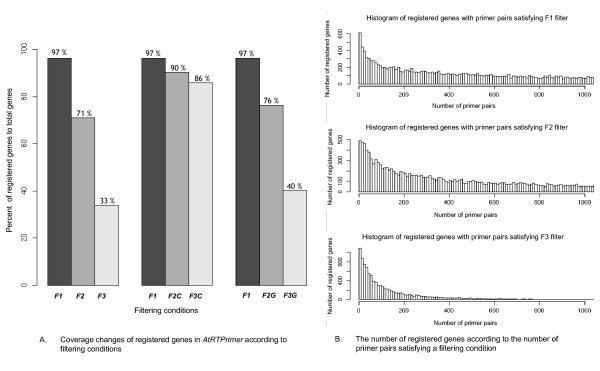
**Statistics in *AtRTPrimer***. Coverage of registered genes in *AtRTPrimer *is presented as the percent of registered genes with primer-pairs satisfying filtering conditions which are denoted as F1, F2, F2C, F2G, F3, F3C and F3G, respectively. (A) Here, the F1 filter has the same filtering condition applied to originally constructed *AtRTPrimer *database. We assume that there are possible amplicons from gDNA or similar cDNA so that we make the following filters: The F2 filter only includes registered genes with primer pairs producing amplicons from gDNA or similar cDNAs, whose sizes are not the same with that of a target gene. We assumed that the sizes of two amplicons are the same when they have the difference of less than five base pairs between each other. The F3 filter only includes registered genes with primer pairs producing no amplicons from both gDNA and similar cDNAs. The F2C and F3C filters have the same filtering conditions as F2 and F3, respectively, except for checking amplicons from only similar cDNAs. The F2G and F3G filters have the same filtering conditions as F2 and F3, respectively, except for considering amplicons from only gDNA. (B) The histograms for the number of registered genes with respect to the number of primer pairs at various filtering conditions are shown. The changes of average numbers are 1,868, 1,212 and 141 primer pairs at filters, F1, F2 and F3, respectively. In the histogram, the number of primer pairs in x-axis is binned with the bin size of 10, and the frequencies of registered genes with more than 1,050 primer pairs in y-axis are omitted.

Approximately 97% of the 28,952 genes investigated were finally registered in *AtRTPrimer *via the following three steps: homogeneity, specificity and construction. To show the coverage of *AtRTPrimer *at various filtering conditions, we calculated the percentage of the number of the registered genes after filtering the 28,952 genes. The coverage of *AtRTPrimer *is maximally decreased from 97% to 33% by the filtering condition based on the additional information on both of similar cDNAs and genomic DNA (leftmost in Figure 2-A).

To probe how much the consideration on genomic DNA contamination affects such a severe coverage change, we applied F2 and F3 filters to the two different types of amplicons separately: one is the amplicons from similar cDNAs and the other from genomic DNA. We found that restricting the possibility of amplification due to genomic DNA contamination made the coverage decrease very sharply (middle in Figure 2-A), while the consideration on amplicons from similar cDNAs affected the coverage change very weakly (rightmost in Figure 2-A). In terms of high coverage, the filtering by the additional information on genomic DNA might not be necessary if gDNA contamination is completely removed in a total RNA in a RT-PCR experiment. However, it is known that it is difficult, especially for plant. Therefore, we believe that this kind of consideration could be useful for a PCR experiment.

We expect that all primer pairs of each registered gene in *AtRTPrimer *are good enough for conventional singleplex RT-PCR without additional filtering. Nevertheless, the *AtRTPrimer *offers user-friendly filtering options to the researcher. For example, the F2 filter can be used for agarose gel-based conventional multiplex RT-PCR experiment in need of primer pairs producing no amplicons with the same sizes from the corresponding genomic DNA and similar cDNAs. In Figure 2-B, there are on average 171 primer pairs per gene registered in the *AtRTPrimer*. In the case of F3 filter, it would be sufficiently stringent for the type of real-time RT-PCR experiment mentioned in Primer bank [5]. A primer pair selected by F3 filter will produce no amplicons from the corresponding genomic DNA and similar cDNAs. The average number of such kind of primer pairs per the registered gene is twenty seven. On the whole, the number of primer pairs of each registered gene after filtering shows that although there is a very large variability between the registered genes, the *AtRTPrimer *appears to have far more informative primer pair resources than any other primer database freely available for Arabidopsis [16], with which researchers can perform the various types of RT-PCR methods (Figure 2-B).

### Evaluation of AtRTPrimer

#### PCR experiments

We performed conventional and real-time PCR experiments under the following conditions: 95°C, 30 sec, 62°C, 1 min, 72°C, 45 sec, 30 cycles. We used total RNAs and genomic DNA of Col-0 as templates for RT-PCR and genomic PCR. These plants were grown under long day condition, and their flowers and inflorescences were harvested at 7 hours after dawn. Total RNA were extracted using RNeasy kit (Qiagen). The cDNA were synthesized with 5 μg of total RNA using RT kit (Promega), and then 20 μl of cDNA were diluted 100 times. Each 1 μl of cDNA were used for conventional and real-time PCR. Real-time PCR were performed using AccuPower Greenstar PCR PreMix (Bioneer) and Exicycler™ Real Time Thermal Block (Bioneer). The AccuPower Greenstar PCR Premix kit consists of Bioneer's Greenstar fluorescent dye and HotStart Taq DNA Polymerase. Amplification plots and melting curves were obtained by companion software in Exicycler™.

#### Evaluation of flowering related genes

**Figure 3:**
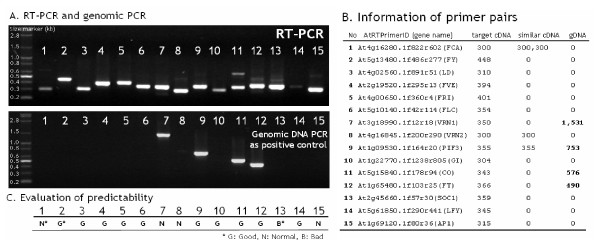
**Results of conventional RT-PCR (A) and genomic PCR (B), and evaluation of *AtRTPrimer's* predictability (C)**. A list of primer pairs of each gene shows AtRTPrimerID, gene name, and expected amplicon sizes from target cDNA, similar cDNA and gDNA (B). The *AtRTPrimer *ID consists of Arabidopsis Genome Initiative (AGI) systemic name, forward primer name and reverse primer name. The selected genes are related to the following flowering pathways[17-19]: long-day photoperiod promotion (GI, CO, FT, SOC1 and PIF3), vernalization (VRN1, VRN2, FRI and FLC), autonomous promotion (LD, FCA, FY and FVE) and floral organ identity (LFY and AP1). We indicate the primer-pairs' predictability (or specificity) of *AtRTPrimer *using the three grades, 'good', 'normal' and 'bad' (C). When one primer-pair produced the amplicons predicted by *AtRTPrimer *as well as the very weak unexpected ones, which are difficult to see in naked eye, we gave 'normal' grade to that primer-pair. The 'good' primer-pairs produced only the amplicons predicted by *AtRTPrimer*. Other case was graded with 'bad'.

**Figure 4:**
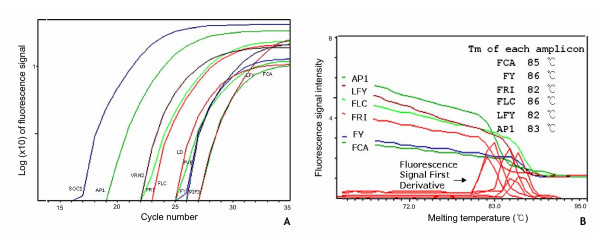
**Results of real-time PCR experiment**. PCR amplification plots for 11 flowering time genes, whose primer pairs have no amplicons from genomic DNA (A) and melting curves of six flowering time genes (B). Each melting temperature of those amplicons is obtained by first derivative of fluorescence signal.

To evaluate the reliability of *AtRTPrimer*, we selected the fifteen target genes from *AtRTPrimer *and performed conventional and real-time PCR under the same PCR conditions (Figure 3 and Figure 4). All the target genes play important roles in multiple pathways of flowering time (Figure 3-B). In case of our test genes, all of similar genes consist of alternative splicing forms to the target genes. In conventional and real-time PCR results, all of target amplicons showed up clearly at the same PCR condition, demonstrating the homogeneity of primer pairs used.

We indicate the primer-pairs' predictability (or specificity) of *AtRTPrimer *using the three grades, 'good', 'normal' and 'bad'. When one primer-pair produced the amplicons predicted by *AtRTPrimer *as well as the very weak unexpected ones, which are difficult to see in naked eye, we gave 'normal' grade to that primer-pair. The 'good' primer-pairs produced only the amplicons predicted by *AtRTPrimer*. If we only count the primer-pairs having 'normal' or 'good' grades in this experiment, the reliability of *AtRTPrimer *is about 93% (Figure 3-C).

Those unexpected amplicons including the very weak ones from cDNA were observed in lane 1, 7, 8, 13 and 15 of RT-PCR (Figure 3-A). In 1, 7, 8 and 15, it is difficult to observe those unexpected bands in naked eye, while it is clear in 13. Although we did not confirm those amplicons by DNA sequencing, we believe that it was from unknown alternative splicing forms. The first reason is that there is not the same size of amplicon from gDNA. Second, In TIGR database, there are only 2,000 genes having alternative splicing variants so far. Therefore, *AtRTPrimer *can not predict those kinds of amplicons if target gene's splicing forms are unknown. A researcher should test a target gene before real experiments to overcome this type of error. In addition, each amplicon in lane 11 and 12 has the same size of amplicon from gDNA as shown in genomic DNA PCR (Figure 3-A). Therefore, we believe that those bands are the amplicons of the contaminated genomic DNA.

We also performed real-time PCR with 11 genes having no amplicons from genomic DNA as predicted by *AtRTPrimer*. The PCR products of target genes have one melting temperature as we observed melting curves (Figure 4-B, other five genes also have one melting temperature; not shown). Typically, it implies that each PCR product has just one amplicon, which in turn indicates the specificity of primer pairs. Therefore, we believe that amplification plots almost reflect the amount of target amplicons from target template (Figure 4-A). However, real-time PCR tends to disregard very weak bands as shown in conventional PCR. In fact, the exact identities of those noisy bands remain unclear until we perform the PCR experiments in various conditions or DNA sequencing. Nonetheless, we believe that our database provides researchers with good starting primer-pairs to perform RT-PCR.

### Web interface of AtRTPrimer

**Figure 5:**
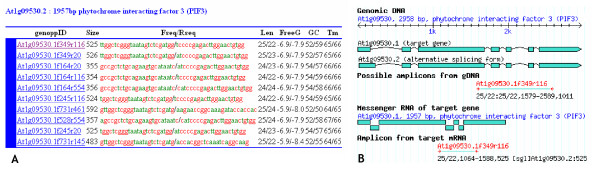
**Web interface of *AtRTPrimer***. Query result and schematic picture of possible amplicons are shown in (A) and (B), respectively. In query result table (A), 'genoppID' is the synonym of AtRTPrimerID and the 'Size' denotes the amplicon length produced by a primer pair. The primer sequences of forward and reverse primer are denoted by 'Fseq' and 'Rseq', respectively. The G or C residues of primer sequences are represented as red colour and the A or T residues as green colour. 'Len' stands for primer length. Here, 'FreeG' are defined as maximal non-specific binding free energy. 'GC' and 'Tm' stand for GC % and melting temperatures of primers. When a user clicks genoppID, a schematic picture shows up as shown in (B). The annotation of a schematic picture is available at Help menu of AtRTPrimer website

We made user-friendly web interface that can be used to retrieve primer-pairs using AGI (Arabidospis Genome Initiative) systemic name or *AtRTPrimer *ID as a single query or a batch of queries, and to show graphically the possible amplicons on a mRNA and a gDNA of a target gene on the fly whenever each primer-pair is clicked (Figure 5). Query result is sequentially ordered by free energy, primer dimerization score, differences of GC and Tm between forward and reverse primers, and amplicon size (Figure 5-A). The schematic picture of possible amplicons will help researchers to interpret their PCR result resulting from pre-designed primer-pair in *AtRTPrimer *(Figure 5-B). We also made web interface for public evaluation of *AtRTPrimer *by world-wide researchers

## Conclusion

We constructed genome-wide homogeneous and specific primer-pair database in Arabidopsis, *AtRTPrimer*, through homogeneity, specificity and construction steps. We also confirmed that *AtRTPrimer *provided a reliable primer pair of a target gene for singleplex PCR experiments except genes having unknown alternative splicing forms. Using *AtRTPrimer*, researchers can properly select pre-designed primer pairs of a target gene for their desired types of RT-PCR by changing the filtering condition as mentioned above. For example, a conventional (agarose gel-based) multiplex PCR requires the different sized amplicons of a set of target genes, so that the researcher should choose pre-designed primer pairs that do not produce other amplicons of the same size compared to the target amplicon. The researcher then selects primer pairs that produce amplicons of different sizes from each other by choosing the following filtering conditions: No same sized amplicons from both a gDNA and similar cDNAs in search condition. To do multiplex PCR, however, in addition to the condition of not having the same sizes of amplicons, there must be another consideration such as minimization of dimerization among primers, intervals between amplicons, and similar expression levels of a set of target genes. In real-time RT-PCR, other amplicons produced by primer dimers, splicing variants and genomic DNA contaminants cause a particularly serious problem for cheapest and most widely used form of real-time RT-PCR as mentioned in PrimerBank [5]. To avoid those problems, researchers can select primer pairs having the only amplicon of a target gene from *AtRTPrimer *by choosing the following filtering conditions: No amplicons of both a gDNA and similar genes in search condition.

We will continue to evaluate *AtRTPrimer *privately and publicly through wet lab collaborating with us as well as world-wide voluntary researchers. In addition, we will update it whenever TIGR release a new version.

## Availability and requirements

This database is available at .

## Abbreviations

**RT-PCR**: reverse transcription polymerase chain reaction, **PA**: Pair-Any score as integer value used in Primer3 program, **PE**: Pair-End score as integer value used in Pimer3 program, **gDNA**: genomic DNA, **cDNA**: complementary DNA to messenger RNA. **AGI**: Arabidopsis Genome Initiative

## Competing interests

The author(s) declare that they have no competing interests.

## Authors' contributions

SH conceived of the study and carried out all of procedures to construct *AtRTPrimer*. DK participated in its design and coordination and helped to draft the manuscript. All authors read and approved the final manuscript.

## Supplementary Material

Additional File 1 Example of how to know possible amplicons from the genomic DNA corresponding with the mRNA of a target gene Click here for file

Additional File 2 Example of how to make a similar gene list of a target gene (A) and how to know possible amplicons from similar genes (B) Click here for file
