# Supplementary material for: AtRTPrimer: database for Arabidopsis genome-wide homogeneous and specific RT-PCR primer-pairs
Source: BMC Bioinformatics. 2006 Mar 30;7:179. doi: 10.1186/1471-2105-7-179 (PMC1524993; doi:10.1186/1471-2105-7-179)
Supplement: Additional File 1 — Example of how to know possible amplicons from the genomic DNA corresponding with the mRNA of a target gene [file 1471-2105-7-179-S1.pdf]

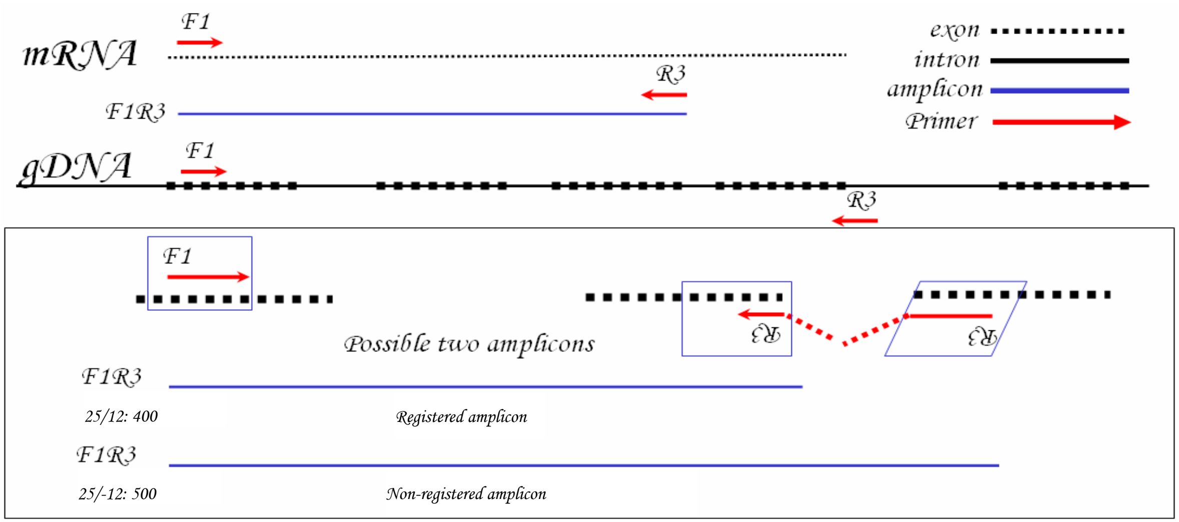


**Figure 1 Example of how to know the possible amplicons from the genomic DNA corresponding with the mRNA of a target gene** As we mentioned in Construction section of the text in the body of article, we used the following terms, ‘gene’, ‘cDNA’ and ‘mRNA’ as the same meaning throughout the text. After filtering out the unqualified primer pairs (Figure 1-A in the body of article), we added the information on possible amplicons from genomic DNA to the qualified primer pair because a total RNA as the template of RT-PCR could be often contaminated by a genomic DNA. Suppose there is one of qualified primer pairs as shown in upper part of figure. Here, we assume that the primer pair of F1 and R3 produce the amplicon of a target mRNA as well as produce amplicons from genomic DNA corresponding to a target mRNA. For R3 primer originated from exon-exon junction, it could bind the two possible regions on genomic DNA shown in low panel. That possibility is denoted as match number at the end of 3’ of a primer to a genomic DNA. The minus symbol denotes no match. For this example, the only one of two different amplicons, however, would be registered in accordance to the following empirical rule: The hybridization of a primer to one exon of two exons in junction would, in most cases, have no specificities for playing a significant role in polymerization. In some case, however, a partial hybridization of a primer to one exon in junction would actually have the specificity for playing a significant role in polymerization. To include amplicons resulting from such the partial hybridization, we accepted the hybridization with exact matches of over ten base pairs containing over one base pair in last three base pairs at the end of 3’. Then, we recoded how long the amplicons of the corresponding genomic DNA is, how many the residues at the end of 3’ end of primer match the corresponding genomic DNA, and whether the amplicon length of a genomic DNA is the same as that of a target gene or not.
