# Supplementary material for: AtRTPrimer: database for Arabidopsis genome-wide homogeneous and specific RT-PCR primer-pairs
Source: BMC Bioinformatics. 2006 Mar 30;7:179. doi: 10.1186/1471-2105-7-179 (PMC1524993; doi:10.1186/1471-2105-7-179)
Supplement: Additional File 2 — Example of how to make a similar gene list of a target gene (A) and how to know possible amplicons from similar genes (B) [file 1471-2105-7-179-S2.pdf]

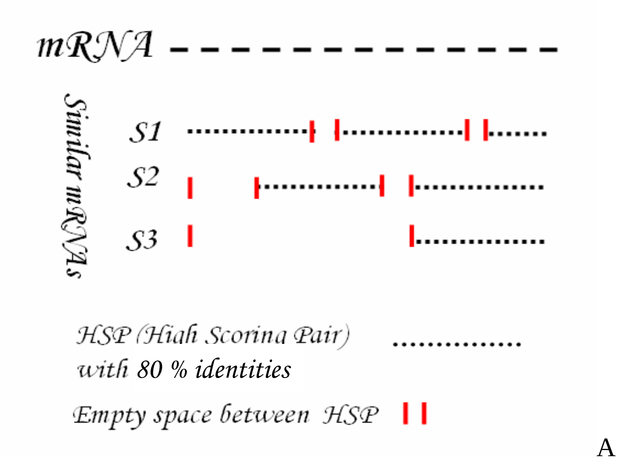

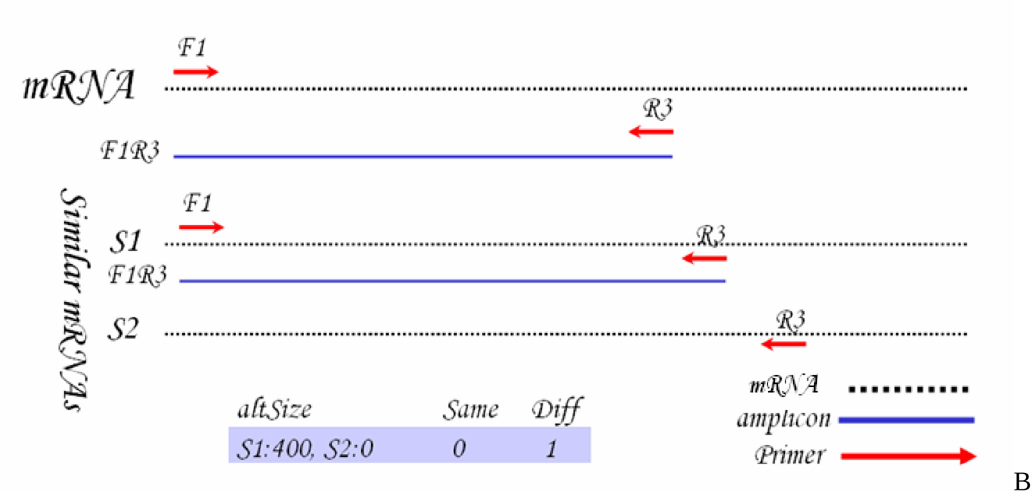

Figure 2 Examples of how to make a similar gene list of a target gene (A) and how to know the possible amplicons from similar genes (B) As we mentioned in Construction section of the text in the body of article, we used the following terms, 'gene', 'cDNA' and 'mRNA' as the same meaning throughout the text. They all indicate unique cDNA sequences. To add the information on possible amplicons from similar cDNAs (or genes or mRNAs), we first defined 'similar genes of a target gene' as genes that have the nucleotide sequence similarity of >80 % to a target gene as well as the total length of empty space between each of a target gene's high scoring pairs (HSPs) with 80 % identities should be smaller than 500 bp. The white spaces between red vertical bars in (A) denote the empty spaces between HSPs. For these lists, we performed BLAST search against 28,592 cDNAs with a target cDNA as query. In case of (A), if there are two similar genes, S1 and S2 satisfying the criteria, the S3 could be excluded because of insufficient HSP length although its' HSP has above 80 % identities. Based on SGL, we can subsequently guess what will be produced from similar genes by a primer pair as shown in (B). In this example, the primer pair, F1 and R3, produces the amplicon, F1R3, from a target gene as well as produce another amplicon, F1R3, from a similar gene, S1. Then, we recoded how long the amplicon of a similar gene is, and whether the amplicon length of a similar gene is the same as that of a target gene or not.
